# Belief in a Just World Decreases Blame for Celebrity Infidelity

**DOI:** 10.3390/bs14100893

**Published:** 2024-10-02

**Authors:** Ching-Yi Huang, Takashi Arai, Tsuneyuki Abe

**Affiliations:** Department of Psychology, Graduate School of Arts and Letters, Tohoku University, Sendai 980-8576, Japan

**Keywords:** adultery, celebrity, flaming, immoral, just-world hypothesis, online firestorms

## Abstract

Justice motivation has been considered one cause of celebrity infidelity scandals becoming flaming incidents. Nevertheless, few studies have examined how the belief in a just world (BJW), one motivation for justice, affects people’s attitudes toward these incidents. This study was conducted to identify the relations among BJW, negative emotions toward infidelity (NE), and celebrity infidelity blaming (CIB). Data were collected from 1186 Japanese adults (mean age = 44.9 years, SD = 13.8; 49.4% male). The results of structural equation modeling revealed that NE positively relates to CIB and plays a mediating role between BJW and CIB. However, BJW neither facilitates NE nor positively predicts CIB; on the contrary, it suppresses both NE and CIB. The findings indicate that BJW decreases people’s blame on celebrity infidelity and suggest future directions for mitigating the issues posed by casting blame on celebrities’ cheating scandals.

## 1. Introduction

Although celebrities’ infidelity (cheating) scandals have long been targets of blaming or criticism, such scandals have attracted much severer blame after the development of the internet, gradually becoming “flaming” incidents in Japan [1,2]. Internet popularity and social networking service (SNS) development in recent years have facilitated communication and the sharing of information among people. They have also brought about many adverse effects such as increased interpersonal conflict among users and the generation of internet “flaming” incidents [3]. The phenomenon of “flaming” (also called online firestorms) on the internet is defined as unspecified internet users blaming a specific target who has violated social norms or who has performed some inappropriate behavior [2,4,5]. These blaming behaviors often engender criminal acts such as defamation and libel. Therefore, they can cause considerable harm and distress to the criticized person. Government data show that flaming incidents in Japan have increased rapidly since 2011 [6], and that they have become a severe social problem today. The behavior of the “flamer” (who writes the blaming comments) infringes on the personal rights of victimized celebrities, who might be, even undeservedly, adversely affected by the severe outcomes of flaming incidents, threatening their mental health and business success. Therefore, to decrease the harm arising from blaming comments, our primary study purpose is to identify individual factors that might contribute to people’s blaming of celebrities involved in infidelity scandals.

### 1.1. Theory and Hypothesis Development

#### 1.1.1. Infidelity

The legal treatment of infidelity has become adjusted over time in Japan. In Japanese criminal law before World War II, infidelity was a criminal act (adultery). After the war, this provision of the criminal code was deleted from criminal law. Today, infidelity is only an issue of civil law between spouses in Japanese society. A person who violates this civil law does not infringe on the public interest. Such a person is only asked to make reparations to the spouse [7]. Therefore, in Japanese society today, infidelity is not a criminal act. However, because the parties engaging in infidelity violate social norms and moral rules [8], infidelity can still be understood as unjust and immoral behavior. An earlier report described that some people who blame a target person do not feel guilty [9], but instead think they are doing a public service to restore justice [1,2,5]. In other words, a blamer is triggered by a justice motivation that is unrelated to a strict legal perspective. Therefore, the leveling of blame at celebrities involved in infidelity scandals should be investigated from a psychological view, with consideration of the effect of “Belief in a Just World” (BJW) [10], one of the justice motivations. Moreover, negative emotions toward infidelity play an important role in explaining blaming behavior. Several studies have pointed out that the negative emotions elicited by immoral incidents (such as infidelity behavior) are also positively related to blaming attitudes [11,12,13]. Consequently, for the current study, we aimed at investigating a sample of Japanese adults to ascertain the relations among BJW, negative emotions toward infidelity, and the blaming of celebrities involved in infidelity.

Several studies of attitudes toward infidelity have indicated different degrees of infidelity tolerance according to demographic characteristics, such as gender and age. Therefore, to prevent these confounding effects from affecting the testing performed for this study, the factors of the participants’ gender and age were controlled when conducting the examination. For example, regarding gender, Buss et al. [14] pointed out that men in the United States, Korea, and Japan are more intolerant of “sexual infidelity,” and that women are more intolerant of “emotional infidelity”. Other studies also presented similar results [15,16,17]. However, one can infer that “sexual infidelity” usually accompanies “emotional infidelity”. Women were found to be more intolerant than men of any type of infidelity [1,8]. Regarding age, Tagler [18] conducted a study of college students and other adults, and the college student group was reported as showing more distress than the adult group after experiencing sexual infidelity. Furthermore, Silva et al. [19] conducted a study with Portuguese people, which revealed that younger participants had more negative attitudes about infidelity than older participants. The findings described above suggest that women and younger people are more intolerant of infidelity, which indicates that they much more readily cast blame for infidelity.

#### 1.1.2. Belief in a Just World and Blaming Attitudes

Belief in a just world, also called just-world belief, holds that people need to believe they live in a just world where good people should be rewarded and bad people should be punished [10,20]. This innate belief was conceptualized as a stable personality characteristic that influences the justice motive [21,22]. It also affects people’s perceptions and judgments of justice [23,24], and is associated with conservative social attitudes and political views [20,25]. When witnessing an unjust incident and finding that victims have experienced some misfortune or harm for no reason, people would feel their beliefs being threatened. To maintain BJW, people will cope with the threat to their beliefs by blaming those victims [10,26,27,28]. Therefore, BJW has been well known as an explanation for victim derogation. In fact, it has led to many related studies conducted to examine this characteristic specifically [26]. However, blaming perpetrators can also be one strategy for maintaining BJW [26]. For instance, Callan et al. [29] pointed out that victim derogation decreases when people find that the perpetrators have been punished. Murayama and Miura [30] used an injury case (a man using a knife to assault others for no reason) as a stimulus to demonstrate that, in the presence of an explicit perpetrator, people preferentially maintain their beliefs through severe punishment of the perpetrator. These findings clearly highlight the characteristics of BJW which affect a person’s tendency to punish wrongdoers to restore justice when a wrongdoer has been identified.

However, it is unclear whether BJWs bring about a blaming attitude toward the perpetrator when the perpetrator is a celebrity. Several Western studies have indicated that a high BJW might provoke people to perceive celebrities positively. For example, McCutcheon [31] reported a positive correlation between BJW and celebrity worship, meaning that people who believe the world is a just place have a much more positive attitude toward the celebrity. Shabahang et al. [32] reported that just-world beliefs are negatively related to celebrity hate. In other words, people with high BJW will regard celebrities as “good people,” which suggests that they believe celebrities deserve well-being, therefore showing much tolerance of celebrities’ behaviors. According to these findings, a high BJW would weaken the blaming attitude when a celebrity perpetrates immoral incident behavior. However, because of the relation with religiosity [33], BJW would exacerbate celebrity blaming behavior, especially in Japan, where Buddhism is a strong influence. Buddhism emphasizes that people who have done bad things should be punished (i.e., Inga-Oho). Even though people with a high BJW regard celebrities as “good people”, people would feel their trust had been betrayed when a celebrity engaged in unjust behavior. Thereby, BJW leads people to need to blame celebrities to restore their own sense of justice. In fact, Murayama and Miura’s [34] finding supported this viewpoint in Japan, which revealed that Japanese people tend to think that wrongdoers with a high social status deserve misfortune, not good fortune. In addition, Nakano [1] pointed out that celebrity infidelity scandals much more easily elicit severe blame in Japanese society than in Western society. According to these findings, we predict that BJW is an inducement to people to cast blame on celebrities involved in infidelity scandals.

#### 1.1.3. Negative Emotions toward Immoral Incidents

Negative emotions, including anger and disgust, are regarded as not only one but as a combination of emotions [35,36,37]. Reports of several studies have described that witnessing someone’s violation of moral values can elicit one’s own negative emotions. According to moral foundation theory [12], for example, people would feel anger or rage when seeing that some harmful or unjust incident has occurred and would feel disgusted when seeing unsanctified incidents. Russell and Giner-Sorolla [38] demonstrated that both anger and disgust are predicted by observing immoral incidents. Oda et al. [13] designed several immoral scenarios as stimuli and confirmed that negative emotions are elicited by immoral incidents.

Earlier studies have also indicated that negative emotions elicited by the immoral incident would trigger people’s tendency to punish that immoral behavior [39]. As the social intuitionist model [12] emphasizes, people intuitively blame a wrongdoer because of their negative emotions when encountering an incident of moral violation. Carlsmith et al. [11] demonstrated that witnessing another person violating moral values would elicit moral outrage and result in a tendency to punish the violators for restoring justice. Furthermore, Olatunji and Puncochar [40] found that moral violations elicit people’s negative emotions, which provokes them to engage in harsher blaming.

Integrating the above findings, and considering that infidelity is regarded as immoral behavior, we predicted that negative emotions are elicited when people confront a celebrity infidelity scandal, and that the emotions affect people by increasing their tendency to cast blame on celebrities involved in infidelity.

#### 1.1.4. BJW, Negative Emotions toward Infidelity, and Celebrity Infidelity Blaming

Regarding BJW as a just motivation that influences people’s perceptions and induces people to behave justly, one can consider that BJW leads to a blaming attitude. On the other hand, the social intuitionist model [12] pointed out that people’s blaming attitudes about incidents of moral violation are affected by negative emotions, which emphasizes that negative emotions lead to moral judgment. Therefore, both BJW and negative emotions tend to affect the intensity of blame that is cast for infidelity incidents. The earlier studies have pointed out the relation of these two aspects. Lerner [41] indicated that the negative emotions’ arousal by the immoral incident is an essential component of a blaming attitude, and that these emotions would be elicited by the need of maintaining BJW. A study by Hafer and Bègue [26] also supports this perception. They summarize several studies and suggest the possibility that the negative emotions of a blaming attitude were derived from BJW. Moreover, the process model of social responses to undesirable events [42] and the justice–injustice judgment model [28] also revealed that emotions play a mediating role in people’s perceptions of experience and the blaming reaction. Consequently, referring to these earlier studies’ results, we predicted that a greater need for maintaining BJW is associated with a greater degree of negative emotions elicited when confronting immoral incidents, and that negative emotions have a mediating effect on a BJW and on celebrity infidelity blaming. In other words, BJW is associated with celebrity infidelity blaming through increased negative emotions.

### 1.2. The Present Study

The aim of the present study is to examine how BJW related to the blaming attitudes about celebrity infidelity scandals felt by people in Japan and to examine negative emotions’ mediating effects. In light of the findings from earlier studies, this study controlled for the confounding effects of gender and age to avoid the effects of participants’ demographic characteristics on BJW prediction. Then, we formulated the following hypotheses.

**Hypothesis 1.**  *BJW is positively related to celebrity infidelity blaming.*

First, several reports of Western studies have described that a high BJW might weaken the blaming attitude when an immoral incident behavior is perpetrated by a celebrity [31,32]. However, considering Japanese culture’s effects (emphasizing that bad people should be punished) and Japanese studies’ findings [1,34], we predicted that people with a strong BJW in Japan feel a need to blame a wrongdoer to restore justice and to maintain their belief, meaning that BJW is positively related to celebrity infidelity blaming.

**Hypothesis 2.**  *BJW is positively related to negative emotions toward infidelity.*

Second, regarding BJW as a justice motivation that drives people’s emotions [26,41], people with a high intensity of BJW are expected to display stronger negative emotions when confronting immoral incidents than people with a low intensity of BJW. Therefore, we predicted that participants who have a high intensity of BJW have higher negative emotions toward infidelity than participants who have a low intensity of BJW.

**Hypothesis 3.**  *Negative emotions toward infidelity are positively related to celebrity infidelity blaming.*

Third, according to several earlier studies, e.g., [12,40], the negative emotions elicited by immoral incidents would lead people to punish a violator. Therefore, we predicted that negative emotions toward infidelity are positively related to celebrity infidelity blaming.

**Hypothesis 4.**  *Negative emotions toward infidelity play a mediating role between BJW and celebrity infidelity blaming.*

Fourth, based on a model used for earlier studies [28,42], we predicted that negative emotions toward infidelity play a mediating role between BJW and celebrity infidelity blaming, which means that BJW is associated with celebrity infidelity blaming through negative emotions.

**Figure 1:**
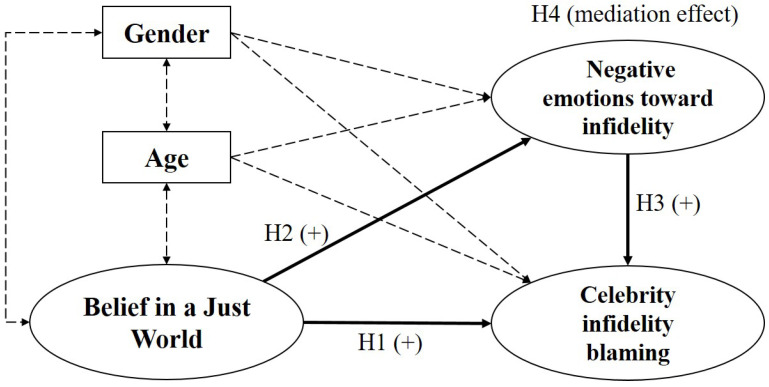
Proposed theoretical model and research hypothesis. (Note. Dotted lines represent control variable relations. Ovals represent latent variables. Squares represent observation variables. H1–H4 = Hypotheses 1–4).

According to these hypotheses, we developed a mediation model using gender and age as control variables. The research model is presented in Figure 1.

## 2. Materials and Methods

### 2.1. Design, Setting, and Participants

This research used a cross-sectional design. We conducted a web-based survey assisted by a Japanese online research company. The participants were selected from a population of Japanese adults, with sampling from 20 groups (2 × 5 × 2) composed by gender (male and female), generation (in their 20s to 60s), and place of residence (metropolitan areas and other areas), to ensure the same homogeneity of respondents’ social backgrounds. All participants were informed about the study before beginning. All acknowledged that their participation in this study was entirely voluntary, that they had the right to withdraw from the process for no reason, that their responses would be handled anonymously, and so on. Informed consent was obtained electronically. Participants received JPY 50 from the research company for participation in the survey. The method of this study was conducted in accordance with the Declaration of Helsinki. Informed consent was obtained from all participants taking part in this study. This study was approved by the Ethical Committee of Graduate School of Arts and Letters, Tohoku University.

Data collection was conducted during 2–3 March 2018. In total, responses from 1240 participants were collected. After excluding participants whose response times deviated significantly from the median (less than half or more than three times the median), 1186 valid questionnaires were retained, resulting in an effective response rate of 95.6%. This sample size exceeds the requirements for structural equation modeling (SEM) and is well suited for conducting SEM analyses [43,44,45,46]. Table 1 presents participant characteristics. The age range was 20–69 years (average age of participants was 44.9 years, SD = 13.8), and 586 (49.4%) were men and 600 (50.6%) were women. There were 592 (49.9%) metropolitan area residents. Among the respondents, 709 (60%) were married and 477 (40%) were single, and 438 (36.9%) were company employees. In addition, 883 participants (75%) often received infidelity scandal information from the internet.

### 2.2. Measures

The measurement materials described below are openly available. More information is presented in the “Data Availability Statement”.

#### 2.2.1. Belief in a Just World Scale (BJW)

The BJW scale we used for this study was modified from Konno and Hori’s [23] scale. This modified scale comprises three items (e.g., “BJW1. In this world, efforts will be rewarded someday”) which the respondents rate using a six-point Likert scale (0 = “strongly disagree” and 5 = “strongly agree”). Cronbach’s alpha coefficient of this scale in the present study was α = 0.62, which indicated the internal consistency of this scale as reliable (>0.60) [47]. The average scores of scale items were used as BJW for later analyses.

#### 2.2.2. Negative Emotions toward Infidelity (NE)

To examine negative emotions toward infidelity, we developed an original scale for this study. According to earlier studies’ finding of the relation between emotion and immoral incidents (e.g., Haidt [12]), we chose the anger and disgust emotions to represent negative emotions to produce the negative emotions scale. These scale items’ descriptions have been adapted from the emotion and arousal checklist (EACL) by Oda et al. [13], which comprised three items. Two items refer to disgust emotions (NE1. Feel disgusted; NE3. Feel filthy). The other item refers to the angry emotion (NE2. Feel angry). Participants first read the vignette: “How do you feel about infidelity in general?” They were then asked to rate their degree of agreement for each item using a six-point scale with possible responses ranging from 0 (I do not agree at all) to 5 (I strongly agree).

#### 2.2.3. Celebrity Infidelity Blaming (CIB)

The celebrity infidelity blaming scale was also developed for this study. This original scale included four items, which comprised two aspects: intolerance from a general and personal moral perspective; each aspect had positive and negative items (e.g., “From common social sense, infidelity is an unforgivable behavior”). To confirm the participant’s attitude toward real “flaming incidents” and to prevent the influence of preferences on specific celebrities, we selected five celebrities whose infidelity scandals had developed into “flaming incidents” as a person of higher social status: a singer (a single female singer who engaged in infidelity with a married singer), a politician (a married woman politician who engaged in infidelity with a married lawyer), a rakugo performer (a married male traditional Japanese comedy performer who engaged in infidelity with a single woman), an actor (a married male actor who engaged in infidelity with a single woman), and an actress (a married actress who engaged in infidelity with a married man). Participants were asked to read “Regarding the [celebrity] infidelity scandal, what is the degree of your agreement toward the following viewpoint?”, and to rate their degree of agreement for each item using a six-point scale, with possible responses ranging from 0 (I do not agree at all.) to 5 (I strongly agree.). The [celebrity] column would fill each flaming incident’s person. All participants were asked to evaluate all the infidelity scandal persons. For analyses, we used the average scores as the respective items’ values.

### 2.3. Statistical Data Analyses

For this study, JASP (Version 0.18.3) was used as the primary tool for data processing. First, to address potential common method bias, Harman’s one-way ANOVA was applied. The results showed that three factors had eigenvalues greater than 1, with the first factor accounting for 41.6% of the variance. This value was below the critical threshold of 50% [48], indicating no considerable common method bias in this research.

Second, we conducted confirmatory factor analysis (CFA) to test composite reliability and construct validity of our measurement, and then conducted structural equation modeling (SEM) to assess the predictive power of a BJW toward celebrity infidelity blaming. Chi-square, the comparative fit index (CFI), the goodness-of-fit index (GFI), the normed fit index (NFI), the standardized root mean squared residual (SRMR), and the root mean square error of approximation (RMSEA) were used to assess the model fit. According to the results of earlier studies [49,50], a CFI, GFI, and NFI larger than 0.90 and SRMR and RMSEA smaller than 0.08 all suggest adequate fit.

## 3. Results

### 3.1. Measurement Model

As a basic examination of the measurement scale used for this study, we conducted a CFA by the entry of all scales (BJW, NE, and CIB). The model fit indices for the CFA model indicated adequate model fit: Chi square (χ^2^) = 231.99, *p* < 0.001, CFI = 0.961, GFI = 0.991, NFI = 0.955, SRMR = 0.039, and RMSEA = 0.074. The construct reliability of NE and CIB for the modified scale was assessed using Cronbach’s α coefficient and composite reliability (CR). Both NE and CIB’s Cronbach’s α are greater than 0.70. All CR values are greater than the suggested values (0.60). Then, following the guidelines provided by Fornell and Larcker [51], the construct validity (including convergent validity and discriminant validity) was assessed using average variance extract (AVE). In terms of the convergent validity, the NE factors had AVE values higher than 0.50, but the CIB factors did not, which indicates 0.49. However, the value of CR higher than 0.60 in the same time indicates that the CIB factor still has adequate convergent validity [51]. In terms of the discriminant validity, the square roots of all factors’ AVE were higher than all the correlation coefficients, respectively, indicating acceptable discriminant validity. In conclusion, the measurement model has good fit. In addition, the NE and CIB scales have adequate composite reliability and construct validity. Table 2 presents all the reliability and construct validity results. We then used these scales to conduct SEM.

### 3.2. Structural Modes and Hypothesis Testing

**Figure 2:**
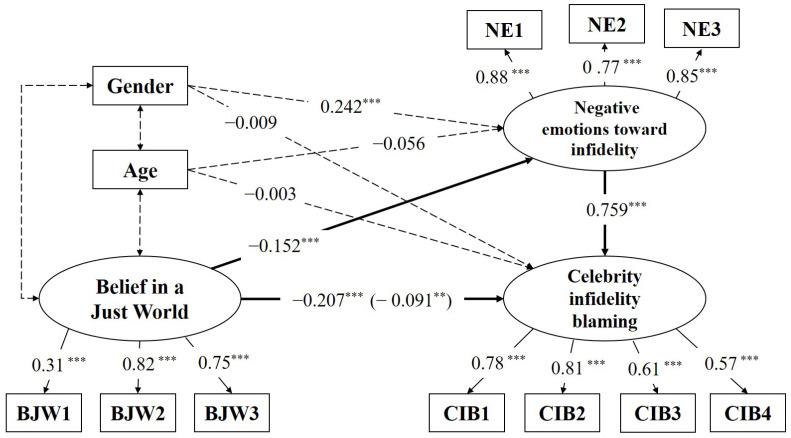
Tested structural equation model. (Notes. *n* = 1186. Dotted lines represent control variable relations. Standardized values are presented. For the path from BJW to CIB, the numbers outside the parentheses represent a direct effect before the NE is added; the numbers inside parentheses represent direct effects after NE is added. BJW = Belief in a Just World, NE = negative emotion toward infidelity, CIB = celebrity infidelity blaming. *** *p* < 0.001, ** *p* < 0.01).

Second, SEM was conducted for the testing of the research model (Figure 1). Referring to earlier findings reported in the literature, we controlled for participants’ gender and age. The results of model fit indices show adequate fit of the structural model: Chi square (χ^2^) = 341.11, *p* < 0.001, CFI = 0.944, GFI = 0.992, NFI = 0.937, SRMR = 0.040, and RMSEA = 0.074. The hypothesis testing results with standardized estimates are shown in Table 3. To examine the direct and indirect effects of BJW and NE, we conducted a bootstrapping method (5000 resampling) by following the suggestions of Efron and Tibshirani [52]. As Table 3 shows, the indirect effect of BJW affecting CIB via NE was found to be significant (indirect effect = −0.116, *p* = 0.000, 95% CI = −0.44 to −0.14). In addition, BJW was found to have a significant direct effect (direct effect = −0.091, *p* = 0.002, 95% CI = −0.41 to −0.06) on CIB, which indicated that the partial mediation effect of NE was supported by the bootstrapping method. However, contrary to the hypotheses, the result of mediation analysis indicated that BJW has a negative relation with NE and CIB. The result is presented in Figure 2.

## 4. Discussion

The primary goal of this study is to use a sample of Japanese adults to examine how BJW is related to blaming attitudes toward celebrity infidelity and to examine the mediating role of negative emotions. Based on theory and findings from earlier research, we predicted that people with a high BJW have more blaming attitudes about celebrities’ infidelity scandals than those with a lower BJW. Moreover, we predicted that negative emotions toward infidelity play a mediating role between BJW and blaming. Four hypotheses were established: H1—BJW is positively related to celebrity infidelity blaming; H2—BJW is positively related to negative emotions toward infidelity; H3—negative emotions toward infidelity are positively related to celebrity infidelity blaming; and H4—negative emotions toward infidelity play a mediating role between BJW and celebrity infidelity blaming. To test our research hypotheses, 1186 Japanese participants’ data were collected. We conducted structural equation modeling using gender and age as control variables to test our theoretical model. The results obtained from statistical data analyses reveal that, even though negative emotions have a mediating effect, BJW neither provoked negative emotions toward infidelity nor provoked people to blame the celebrity’s infidelity scandal.

### 4.1. Theoretical Contributions

First, this study revealed how BJW affects celebrity infidelity blaming. Most earlier studies have used simulated scenarios as valuation objects. However, using scenarios has been pointed out as presenting unrealistic problems that are irrelevant to the participants’ world, which would less threaten the need to BJW [26]. To reduce this shortcoming, this study instead selected actual celebrity infidelity scandals, which had become flaming incidents in Japan, as valuation objects. Therefore, our findings reflect the actual BJW effects on celebrity infidelity blaming more reliably than other earlier studies’ findings.

Second, this study uncovers a new consequence of BJW by revealing its negative relation with casting blame on celebrities involved in infidelity. Even though several earlier studies of BJW have pointed out that immoral incidents threaten people’s BJW, which leads people to blame wrongdoers and thereby maintain their BJW [10,23,26,30], few studies have examined whether the social status of the wrongdoer causes the same effect or not. Considering the disparate findings reported from Western and Japanese studies [1,30,31,32,34], we decided to investigate the belief in a just world (BJW) effect, using celebrity infidelity scandals as our evaluation subjects. Although the analysis results contradict our hypothesis (Hypothesis 1), they are still consistent with the findings from Western studies [31,32]. According to those studies’ statements, the reason BJW weakens the blaming attitude toward celebrities is that people with a high intensity of BJW would regard celebrities as “good people” who deserve well-being. Thereby, they would show much tolerance of celebrities’ behaviors. By contrast, our findings show the opposite result from the Japanese study [30,34], which might be attributable to the different types of evaluated immoral behavior. Murayama and Miura [34] designed a scenario about a person with a high social status stealing another person’s property. This behavior did not merely violate moral rules; it was regarded as a criminal act, which led people with a high BJW to punish them for restoring justice (criminal should be punished). However, as the Section 1 of this paper presents, today in Japan, even though infidelity behavior violates moral rules, it is not regarded as a crime. It is regarded merely as a private affair among the parties concerned. It has no relevance to the public interest. Consequently, people with a highly intense BJW would minimize or deny the need for justice restoration, thereby decreasing the blaming of the parties involved in infidelity.

Third, this study further explores whether BJW does not provoke negative emotions toward infidelity. By regarding BJW as a justice motivation that influences people’s perceptions and induces people to behave justly, we predicted that people with a high BJW would display stronger negative emotions when confronting immoral incidents (Hypothesis 2). However, our finding revealed that BJW has a negative relation with negative emotions toward infidelity, which indicated the opposite result. The possible explanation is, as described in the paragraph above, that infidelity in the legal view is not to be regarded as a crime but as a private affair in Japan, which leads people with a high intensity of BJW to consider such behavior as not so severe in many varieties of moral violations, thereby decreasing the intensity of negative emotions toward infidelity.

Another explanation of the negative relation between BJW and both NE and CIB is that BJW is not unidimensional but bidimensional [34,53]. Each dimension of BJW has different effects on blame attitudes in unjust incidents. According to Murayama and Miura [34], there are two types of BJW: Belief in Immanent Justice (BIJ) and Belief in Ultimate Justice (BUJ). Their research results indicated that BIJ promotes a tendency to punish perpetrators harshly, while BUJ leads people to create psychological distance between the victim and themselves. Based on these findings, the negative relation between BJW and both NE and CIB might be attributable to the effects of BUJ in our research. However, we were unable to confirm this point in the current study because we did not measure BIJ and BUJ directly. Nevertheless, this speculation is worth further exploration in future research to verify its potential.

Fourth, Hypothesis 4 was supported. The results demonstrate that NE have a mediating effect in relation between BJW and CIB. Both direct and indirect effects were found to have significant negative relations. This finding indicates that even when mediated by NE, people with a weak BJW would blame the celebrities’ infidelity more than those with a strong BJW.

Finally, the SEM results revealed that the gender variable has a significant positive relation with the NE variable, indicating that women are more likely than men to have negative emotions toward infidelity, which is consistent with the findings of earlier studies [1,8]. However, for gender and age variables, no significant relation with CIB was found, which contradicts the findings of some earlier studies of infidelity (e.g., [17,19]). These inconsistencies might be attributable to those studies’ different research designs. In other words, the earlier research design did not specifically examine the influence of BJW but also did not consider the social status of the individuals involved in the infidelity as an essential factor. The moral standard of emphasis on trust and respect for individuals with a high social status in Eastern culture [12] might engender more aligned evaluations of high social status individuals’ behaviors by different genders and age groups. Therefore, no significant differences were found in the current study. The explanations above suggest that it is more important to control for the effects of gender than age when examining the issues related to negative emotions about infidelity. However, especially in Eastern culture, when considering infidelity behavior involving high social status individuals, both gender and age apparently cease to be important factors.

### 4.2. Implications for Practice

As one of many flaming incidents in modern Japanese society, celebrities involved in infidelity scandals have caught much attention and have been adversely affected by flamers’ criticisms. Considering this background, reducing celebrity infidelity incidents as flaming targets is an important issue. Recently, the Japanese legislature increased criminal liability for defamation and libel, aiming at a reduction in flaming incidents. However, a flamer’s blaming behavior originates in justice motivation, not from a legal view. Therefore, to find a method to mitigate these flaming difficulties, it is necessary to identify how BJW contributes to people’s blaming attitudes toward celebrities’ infidelity scandals.

Based on our findings, to prevent celebrity infidelity scandals from becoming flaming incidents, researchers should devote attention to how to elicit internet or SNS users’ BJW, and decrease people’s negative emotions about celebrity infidelity. For example, to elicit BJW, one specific measure could refer to some websites and SNSs that have already provided reminders before users post comments (e.g., the user should follow the law; offenders will be liable to punishment). It can also add some reminders, such as “People will get what they deserve by legal treatment; there is no need to use any offensive comment to restore justice”, to help elicit users’ BJW. On the other hand, to decrease the effects of negative emotions, restrictions on instant reply or share functions would be helpful. This function prevents users from typing comments immediately, which lets users have time to calm down and not be triggered by the negative emotions elicited by a celebrity infidelity scandal. In conclusion, the findings of our study provide future directions for mitigating the difficulties posed by casting blame on celebrities involved in infidelity. However, whether the specific approaches described above are effective at achieving the mitigation function remains to be examined through future research.

### 4.3. Limitations and Future Directions

This study has several limitations and restrictions. First, because the definitions used for this study (e.g., infidelity) were set as related to Japanese laws and social culture, this study’s findings might not be generalizable to non-Japanese populations. Therefore, cross-cultural comparisons of the results obtained from this study must be verified further. Future research should specifically examine the substantiation and replication of these results in other countries.

Second, the participants’ age and marital status must be noted. Even though we controlled the age variable and found no significant effect, the mean age (44.9 years) of this study’s participants remained somewhat high. Moreover, approximately 60% of participants were married at the time of the survey. Considering that our examination was related to infidelity behavior, differences in marital status might have influenced participants’ attitudes, although our test was unable to evaluate this effect. Therefore, future studies should specifically examine other groups of younger people and consider the participants’ marital status when sampling for additional tests.

Third, given that the study participants were Japanese, we used the modified Japanese BJW scale from Konno and Hori [23]. However, the Cronbach’s alpha coefficient of this BJW scale in our study was somewhat low. Considering that this scale was developed in 1998, it might be outdated today. Moreover, as we mention in the discussion chapter, several studies have indicated that BJW is not unidimensional but bidimensional (e.g., [53]), with different effects for blame attitudes in unjust incidents. Given these issues, future studies should use more reliable BJW scales to conduct further tests.

Finally, the cross-sectional nature of this study limits our ability to infer causal relations. Future research should examine the direction of the relation between BJW, NE, and CIB in greater detail using a longitudinal or experimental research design.

## 5. Conclusions

This study was conducted to identify the relation between belief in a just world (BJW), negative emotions toward infidelity (NE), and celebrity infidelity blaming (CIB). The results indicate NE as positively related to CIB and as having a mediating effect in the relation between BJW and CIB. However, BJW neither facilitates NE nor positively predicts CIB; on the contrary, it suppresses both NE and CIB. Consequently, the findings revealed that BJW decreases people’s blaming attitude toward celebrities involved in infidelity. The researchers should devote attention to how to elicit internet or SNS users’ BJW and decrease their negative emotions to prevent celebrity infidelity from becoming a flaming incident in the future.

## Figures and Tables

**Table 1:** Characteristics of participants.

Personal Characteristics		*n*	%
Gender	Male	586	49.4
	Female	600	50.6
Age	20s	239	20
	30s	234	20
	40s	236	20
	50s	237	20
	60s	240	20
Marital status	single	477	40
	married	709	60
Received celebrity infidelity information from the internet	never	97	8
	rarely	84	7
	sometimes	122	10
	often	221	19
	usually	273	23
	always	389	33
Residence	metropolitan area (incl. Kanto and Kansai)	592	49.9
	other provinces	594	50.1
Occupation	public servant	48	4
	company manager	12	1
	company employee	438	36.9
	independent business	95	8
	part-time job	158	13.3
	full-time homemaker	243	20.5
	student	30	2.5
	jobless	115	9.7
	others	47	4

Note: *n* = 1186.

**Table 2:** Confirmatory factor model fit and reliability, construct validity assessment.

Factor	Item	*M*	*SD*	Factor Loading	α	CR	AVE	Correlation Matrix	
								BJW	NE
BJW	BJW1	2.13	1.29	0.31	0.62	0.68	0.44	(0.66)	
	BJW2	1.25	1.07	0.82					
	BJW3	1.18	1.06	0.75					
NE	NE1	2.90	1.48	0.88	0.87	0.87	0.70	−0.15 ***	(0.83)
	NE2	2.18	1.51	0.77					
	NE3	2.85	1.49	0.85					
CIB	CIB1	2.91	1.19	0.78	0.81	0.79	0.49	−0.21 ***	0.77 ***
	CIB2	2.15	1.22	0.81					
	CIB3	3.20	1.19	0.61					
	CIB4	3.15	1.07	0.57					

Model fit statistics: Chi square (χ^2^) = 231.99, *p* < 0.001, CFI = 0.961, GFI = 0.991, NFI = 0.955, SRMR = 0.039, and RMSEA = 0.074. Note: original item descriptions all written in Japanese. *** *p* < 0.001; α = Cronbach’s α coefficient; CR = composite reliability; AVE = average variance extracted. The values given in parentheses are the square roots of the AVE. BJW = belief in a just world; NE = negative emotions toward infidelity; CIB = celebrity infidelity blaming.

**Table 3:** Hypothesis testing result for the mediation model.

Effect	Relation	Standardized Estimates	Confidence Interval 95%		*p*-Value	Result
			Low	High		
Control variables	Gender → NE	0.242	0.466	0.780	0.000	
	Gender → CIB	−0.009	−0.109	0.077	0.732	
	Age → NE	−0.056	−0.011	0.000	0.062	
	Age → CIB	−0.003	−0.004	0.003	0.905	
Direct effect	H1: BJW → CIB	−0.091	−0.406	−0.064	0.002	Not supported
	H2: BJW → NE	−0.152	−0.798	−0.258	0.000	Not supported
	H3: NE → CIB	0.759	0.496	0.599	0.000	supported
Indirect effect	H4: BJW → NE → CIB	−0.116	−0.439	−0.139	0.000	supported
Total effect	H4: BJW → CIB	−0.207	−0.758	−0.279	0.000	

Model fit statistics: Chi square (χ^2^) = 341.11, *p* < 0.001, CFI = 0.944, GFI = 0.992, NFI = 0.937, SRMR = 0.040, and RMSEA = 0.074. Note: test for mediation using a bootstrap analysis with a 95% confidence interval. Bootstrap sample = 5000 with replacement. H1–H4 = Hypotheses 1–4. Gender: 0 = male, 1 = female. BJW = belief in a just world; NE = negative emotions toward infidelity; CIB = celebrity infidelity blaming.

## Data Availability

The data described in this article and the entire measurement materials are openly available from the Open Science Framework at: https://osf.io/y2xd5/?view_only=87bef51bb6234a73bc5d602f1be2078f (accessed on 24 June 2024).
